# Therapeutic Magnetic Resonance (TMR) in Regenerative Medicine: In Vitro Study to Support Future Clinical Applications

**DOI:** 10.3390/jfb17080356

**Published:** 2026-07-24

**Authors:** Micaela Berni, Laura Caliogna, Elisa Lenta, Gloria Acquafredda, Chiara Valsecchi, Stefania Croce, Sara Bozzini, Patrizia Comoli, Mario Mosconi, Gianluigi Pasta, Maria Antonietta Avanzini, Mirko Belliato

**Affiliations:** 1 Orthopedics and Traumatology Clinic, IRCCS Policlinico San Matteo Foundation, 27100 Pavia, Italy; m.berni@smatteo.pv.it (M.B.); mario.mosconi@unipv.it (M.M.); g.pasta@smatteo.pv.it (G.P.); 2 Cell Factory, Department of Mother and Child Health, Fondazione IRCCS Policlinico San Matteo, 27100 Pavia, Italy; e.lenta@smatteo.pv.it (E.L.); c.valsecchi@smatteo.pv.it (C.V.); s.croce@smatteo.pv.it (S.C.); p.comoli@smatteo.pv.it (P.C.); ma.avanzini@smatteo.pv.it (M.A.A.); 3 Paediatric Haematology and Oncology, Department of Mother and Child Health, Fondazione IRCCS Policlinico San Matteo, 27100 Pavia, Italy; g.acquafredda@smatteo.pv.it; 4 Immunohaematology and Transfusion Service, Cell Manipulation Laboratory, Fondazione IRCCS Policlinico San Matteo, 27100 Pavia, Italy; s.bozzini@smatteo.pv.it; 5 Department of Clinical, Surgical, Diagnostic and Pediatric Sciences, University of Pavia, 27100 Pavia, Italy; 6 Department of Intensive Medicine, Fondazione IRCCS Policlinico San Matteo, 27100 Pavia, Italy; m.belliato@smatteo.pv.it

**Keywords:** TMR^®^, bone marrow-derived MSCs, adipose-derived MSCs, bone healing

## Abstract

This *in vitro* study investigates the effects of therapeutic magnetic resonance (TMR^®^), a novel biophysical stimulation technology, on mesenchymal stromal cells (MSCs) to support its potential application in regenerative medicine. The role of pulsed electromagnetic fields in bone healing is already established. We evaluated whether TMR^®^ influences MSC proliferation, differentiation, and immunomodulatory properties *in vitro*. Bone marrow-derived MSCs (BM-MSCs) and adipose-derived MSCs (AD-MSCs) were cultured with or without TMR^®^ exposure and assessed through flow cytometry, karyotype analysis, senescence assays, proliferation tests, gene expression analysis, and osteogenic differentiation assays. TMR^®^ did not alter MSC phenotype, proliferation, senescence, or genomic stability, confirming its safety profile. Notably, treated MSCs showed enhanced osteogenic differentiation, with increased early expression of key osteogenic markers (RUNX2, ALP, and COL1A1) and greater collagen deposition compared to untreated controls. TMR^®^ reduced peripheral blood mononuclear cell proliferation and MSC ROS production, suggesting anti-inflammatory and antioxidative effects. Overall, TMR^®^ appears to be a safe, non-invasive stimulus able to promote osteogenic differentiation, supporting its potential clinical application in bone regeneration. Further *in vitro* studies and clinical trials are needed to confirm these findings.

## 1. Introduction

The ability of electromagnetic fields to positively influence osteogenesis was first hypothesized in the late 1950s, as a result of the discovery of the piezoelectric properties of bone by Fukada and Yasuda [1]. In the following years, other scientists, including Shamos and Levine, demonstrated that all biological tissues are able to develop electrostatic charges when subjected to mechanical stress, and that increased bone matrix production was associated with these charges [2].

This observation suggested the possibility of applying electrical energy to modify bone cell behavior [3]. In this context, pulsed electromagnetic fields (PEMFs) acquired particular interest. PEMFs are low-frequency magnetic fields with a specific waveform and constant amplitude variation. These fields are generated by an alternating current passing through a coil. In 1979, they were approved by the FDA as a safe and effective treatment for bone fracture healing, non-union, congenital pseudoarthrosis, and failed spinal fusions [4,5,6].

It is well known that PEMFs interact with several biological systems at the molecular, cellular, and tissue levels, affecting enzyme kinetics and signaling pathways. They cause cell membrane depolarization, repolarization, and hyperpolarization. This electrical shift transiently opens voltage-gated Ca^2+^ and Na^+^ channels. The increased intracellular ion concentration activates enzymes such as protein kinase C and the phosphatase calcineurin. The cells generate second messengers, such as cAMP and cGMP, driving downstream signaling pathways that, through transcription factors, lead to the gene upregulation responsible for cell survival and differentiation (such as RUNX2, DLX5, and BMP2). Ultimately, the cell increases its production of regenerative proteins and growth factors, restoring tissue homeostasis [7,8].

Nowadays, therapeutic parameters like frequency, intensity, and exposure time are not strongly supported by human long-term clinical trials required to optimize parameters and understand mechanisms of action. Nevertheless, given the great relevance of physical therapies in regenerative medicine, their clinical application could be associated with specific limitations and potential side effects, although in vitro studies have not reported cytotoxicity or genotoxicity [9,10,11,12,13,14].

Several studies show that PEMFs can interact with each phase of the physiological healing process of bone fractures. It is demonstrated that during the bone formation phase, recruited mesenchymal stromal cells (MSCs) begin to differentiate into osteoblasts or chondrocytes under the influence of Runx-2 and Osx transcription factors [15].

MSCs are adult multipotent stem cells, characterized by plastic adhesion, fibroblast-like morphology, and the ability to give rise to colony-forming units (CFU). MSCs were first identified by Friedenstein in human bone marrow [16], following studies that described adipose tissue, placenta, cord blood, and fetal tissues as further sources to isolate and expand in vitro MSCs. As defined by Dominici et al. [17], MSCs do not express a unique cell surface marker, but they are identified by the simultaneous presence of CD90, CD105, CD73, and the lack of CD34, CD45, CD19, and HLA class I. One of MSCs’s hallmarks is their multipotency, defined as the ability to differentiate in vitro into bone, cartilage, and adipose tissue. The high proliferative capacity, the homing ability, the differentiation potential, and the low immunogenicity make MSCs good candidates for several clinical applications. For their regenerative and immunomodulatory properties, they have acquired great interest for therapies targeting skeletal injuries, autoimmune diseases, and other immune-mediated conditions. Differences in behavior and applications are related to the MSC source. Adipose-derived MSCs (AD-MSCs) have higher regenerative potential and are easier to obtain compared to bone marrow-derived MSCs (BM-MSCs); on the other hand, BM-MSCs have shown higher differentiation potential than ASCs [18]. Culture conditions, such as medium composition, seeding density, viability, temperature, gas concentrations, and physical or chemical stimulants/treatments, are reported to affect MSC effects and potency, determining different qualitative and quantitative outcomes if applied to a specific clinical setting.

Recently, a new technology has been developed in this field that uses three specific biophysical frequency ranges, the only ones necessary and scientifically validated, to act directly on the bioelectric component of the cell (TMR^®^). To our knowledge, little is known about the TMR mechanism that mediates cell biology [19].

Therefore, in this *in vitro* study, we aimed to investigate the role of TMR treatment on MSC differentiation towards bone, in particular to define “safety” and “efficacy” for its application both *in vitro* and *in vivo*.

## 2. Materials and Methods

### 2.1. Therapeutic Magnetic Resonance

Therapeutic Magnetic Resonance (TMR^®^) is a biophysical technology (Diapason, Thereson, Rome, Italy) that acts on the bioelectric component of the cell, restoring its proper functioning. The low-intensity magnetic field has no direct therapeutic purpose but allows the frequencies to penetrate up to 30 cm into the tissues, reaching deep into the areas to be treated. TMR^®^ is characterized by specific waveforms, frequencies, packaging, intensity, and antennas to deliver a group of three frequencies simultaneously with two specific programs: vascular (program 6) and osteoarticular (program 8), combined to obtain regenerative, anti-inflammatory, and anti-oxidative effects (program 6–8). The range of frequencies is in three specific biological settings: 0.1–3.0 Hz (vasomotion and vascular stimulation), 10.0–30.0 Hz (osteoarticular and musculoskeletal stimulation), and 200.0–250.0 Hz (central and peripheral nervous system stimulation), and packed contemporaneously together.

### 2.2. Mesenchymal Stromal Cells

#### 2.2.1. Bone-Marrow-Derived MSCs (BM-MSCs)

We considered BM-MSCs previously expanded and cryopreserved at −196 °C in vitality. BM-MSCs were obtained from leftover BM samples of 4 healthy donors, after obtaining written informed consent and Ethical Committee approval (protocol number 0061549/22 of 11 January 2024). BM mononuclear cells, obtaining by BM gradient separation (Lympholyte, Cedarlane, Milan, Italy), were plated at 160,000 cells/cm^2^ in complete medium: Dulbecco’s Modified Eagle Medium (DMEM) low glucose (Gibco, Milan, Italy) 5% platelet lysate (PL) (Macopharma, Tourcoing, France), 50 μg/mL streptomycin, 50 U/mL penicillin, and 2 mM L-glutamine (Euroclone, Milan, Italy) and incubated at 37 °C 5% CO_2_. The complete medium was changed two times a week; at confluence ≥ 90%, MSCs were detached with TrypLE (Gibco) and replated at 4000 cells/cm^2^. These passages were repeated to reach P4. At each passage, MSCs were cryopreserved for experiments. At the time of use, MSCs were thawed at 37 °C and plated in complete culture medium.

#### 2.2.2. Human Adipose-Derived Stem Cells (AD-MSCs)

Two different AD-MSC lines were purchased from the CliniSciences Company (catalogue number CTICC1.9 and lot number SK0595, Guidonia di Montecelio, Italy). The cells were cryopreserved and after thawing were cultured at the concentration of 3000/cm^2^ in DMEM F12-HAM (Sigma-Aldrich, St. Louis, MO, USA) supplemented with 100 μg/mL streptomycin, 100 U/mL penicillin, 0.25 μg/mL amphotericin, and 10% FBS (Fetal Bovine Serum, Bernolshim, France) in a humidified atmosphere, 5% CO_2,_ at 37 °C up to 95% confluence. The adherent cells were detached with Trypsin 0.05%/EDTA 0.02% (PAN-biotech, Aidenbach, Germany) and cultured in a new flask.

### 2.3. Cell Seeding and TMR Treatment

BM-MSC and AD-MSC lines were seeded in two different 12-well plates at a density of 10,000 cells/well in complete medium or in osteogenic medium (StemPro™ Osteogenesis differentiation kit, Thermo Fisher Scientific, Waltham, MA, USA). One control plate was cultured without PEMF treatment (not-treated, NT); the other was exposed to TMR (treated, T) at a frequency between 0.1 and 250 Hz for 16 min every 6 h, 4 times a day. These parameters were defined to comply with standard acute care therapy. The antennas used for cell culture are the same in type, shape, size, and number of turns as those used in commercial devices but encapsulated in a plastic disk to allow use in a CO_2_ incubator. These antennas were put into the CO_2_ incubator directly linked to the device, out of the incubator. The plates were maintained for the entire time the culture was on the antennas.

### 2.4. MSC Characterization

#### 2.4.1. Flow Cytometry

Analysis of cell-surface markers was performed by flow cytometry (FACS Canto II, Becton Dickinson BD, Milan, Italy) with monoclonal antibodies against CD73, CD34, CD90, CD14, CD45, CD105, and HLA-DR (BD). Appropriate isotype-matched antibodies were employed as controls. Analysis was performed by FACSDiva™ Software Version 4.1 (BD).

#### 2.4.2. Cytogenetic Analysis by Conventional Karyotype

BM-MSCs and AD-MSCs exposed or not to TMR^®^ were maintained in DMEM without overnight PL in order to obtain cell synchronization. After 29 h of culture with complete medium, colcemid (IrvineScientific, Santa Ana, CA, USA) was added at 1 μg/mL and incubated for 4 h at room temperature. The cells were then fixed and spread according to standard procedures. Metaphases of cells were GTG-banded and karyotyped in accordance with the International System for Human Cytogenetic Nomenclature (ISCN) [20]. At least 20 metaphases were analyzed for each sample.

#### 2.4.3. Senescence Assay

MSC senescence was evaluated on TMR-exposed cells by Senescence Kit (Cell Signaling Technology, Danvers, MA, USA) and compared to non-exposed cells. MSCs were expanded until senescence was reached, defined as the passage when the number of cells recovered ≤ the number of cells plated. At this time 2500 cells/well were plated in culture medium at 37 °C, 5%CO_2_ for 24 h. After washing and fixation, β-galactosidase staining solution was added, and the plate was incubated overnight at 37 °C in a dry incubator. The senescent cells were colored in blue under a phase contrast microscope (4×).

### 2.5. Proliferation Assay (Alamar Blue)

To evaluate MSC cell proliferation, optical density was measured at 3, 7, 10, and 14 days of culture using Alamar Blue (Thermo Fisher Scientific, Milano, Italy) according to the manufacturer’s instructions.

### 2.6. Real-Time Quantitative Polymerase Chain Reaction (qPCR)

To evaluate the gene expression during *in vitro* osteogenic differentiation, we tested *col1a1*, *alp*, and *runx2* expression at 3, 7, and 14 days. The total RNA was extracted from monolayer culture using the Gene MATRIX RNA purification kit (Biosigma, Cona, Italy) according to the manufacturer’s instructions and reverse-transcribed into cDNA using random primers and M-MLV Reverse Transcriptase (Promega, Milano, Italy). Quantitative real-time PCR (qRT-PCR) was performed in triplicate using primers from Qiagen (Qiagen, Hilden, Germany) reported in Table 1 and Quantifast-SYBR Green PCR Kit (Qiagen, Hilden, Germany) and StepOnePlus™ Real-Time PCR System (Applied Biosystems™, Thermo Fisher Scientific, Waltham, MA, USA), according to the manufacturer’s instructions. The housekeeping gene expression *β2M* (β2-microglobulin gene, Qiagen, Hilden, Germany) was used to normalize the qPCR results. The gene expression of differentiated cells was reported as fold change versus expression of MSCs cultured in a complete culture medium as described above (control).

### 2.7. Immunofluorescence Assay

The presence of osteogenic matrix deposition was evaluated by immunofluorescence assay after staining with phalloidin and COL1A1. Cells were cultured on transparent microscope coverslip slides (Size: Round Diameter 10 mm). At 21 days after differentiation induction, samples were fixed with 4% formaldehyde solution (Sigma-Aldrich, MO, USA) at 4% in PBS for 30 min. After washing with PBS, cells were permeabilized with 0.4% Triton X-100 (Sigma-Aldrich) and then incubated overnight at 4 °C with anti-COL1A1 (PA1-26204, Thermo Fisher Scientific, Waltham, MA, USA). The day after, samples were incubated with anti-rabbit IgG (A-21207, Thermo Fisher Scientific) for 30 min at room temperature. After washing, the slides were incubated with PHALLOIDIN for 30 min (PHALLOIDIN Atto 488 Sigma) and nuclei stained with DAPI (ProLong™ Gold Antifade Mountant, Thermo Fisher Scientific). The images were acquired on a fluorescence microscope (Thunder Leica, Wetzlar, Germany).

### 2.8. Histological Staining

10,000 BM-MSCs and AD-MSCs exposed or not to TMR were cultured in a 24-well plate, in the presence of differentiation medium (StemPro™ Osteogenesis differentiation kit, Thermo Fisher Scientific) for 7, 14, and 21 days. At these time points, the wells were stained for calcium deposition with Alizarin Red S (Sigma-Aldrich) to detect osteogenic differentiation.

### 2.9. In Vitro PBMC Proliferation Assay

Peripheral blood mononuclear cells (PBMCs) were obtained by gradient separation (Lympholyte, Cedarlane, Burlington, NC, USA) from 10 healthy donors’ heparinized peripheral blood samples after obtaining written informed consent and Ethical Committee approval (protocol number 14373/23 of 7 March 2023). Proliferation assay was performed in triplicate in flat-bottomed 96-well tissue culture plates (BD, Milan, Italy) in the presence or absence of MSCs as previously reported [21]. Briefly, MSCs were seeded at MSC: PBMC ratios of 1:2, 1:20 and 1:200 per well and allowed to adhere overnight before adding 1 × 10^5^ PBMCs per well with or without phytohemagglutinin (PHA, Sigma-Aldrich, 4 μg/mL). TMR-exposed or non-exposed cultures were maintained at 37°, 5% CO_2_. After 48 h ^3^H-thymidine (37 kBq/well, specific activity 248 GBq/mmol, PerkinElmer, Waltham, MA, USA) was added and harvested after 18 h. ^3^H-thymidine incorporation was measured by Topcount (PerkinElmer, Milan, Italy); results were expressed as a stimulation index (SI = cpm stimulated/cpm unstimulated).

### 2.10. Reactive Oxygen Species (ROS) Evaluation

ROS production in BM-MSCs and AD-MSCs exposed and non-exposed to TMR was detected using the Total Reactive Oxygen Species Assay Kit (Invitrogen, Milan, Italy). Briefly, 2.5 × 10^5^ MSCs were incubated with 1 × FITC-conjugated ROS Assay Stain Solution for 1 h at 37 °C with 5% CO_2_. After washing, 10,000 events were acquired using a Canto II (BD) flow cytometer. The data were analyzed by Kaluza Software Version 2.4 (Beckman Coulter, Milan, Italy) and results expressed as Mean Fluorescence Intensity (MFI) [22].

### 2.11. Statistical Analysis

All the statistical analyses were performed with GraphPad Prism 8.4.2. All the data were tested for normality and subsequently analyzed with paired *t*-tests, a one-way ANOVA followed by Tukey’s and Dunnett’s tests for multiple comparisons, or by simple linear regression. The differences were considered statistically significant when *p* < 0.05.

## 3. Results

### 3.1. MSC Characterization

MSCs were tested by flow cytometry to verify their typical cell surface antigen expression. Exposure to TMR^®^ did not influence the expression of MSC markers (Table 2).

**Figure 1:**
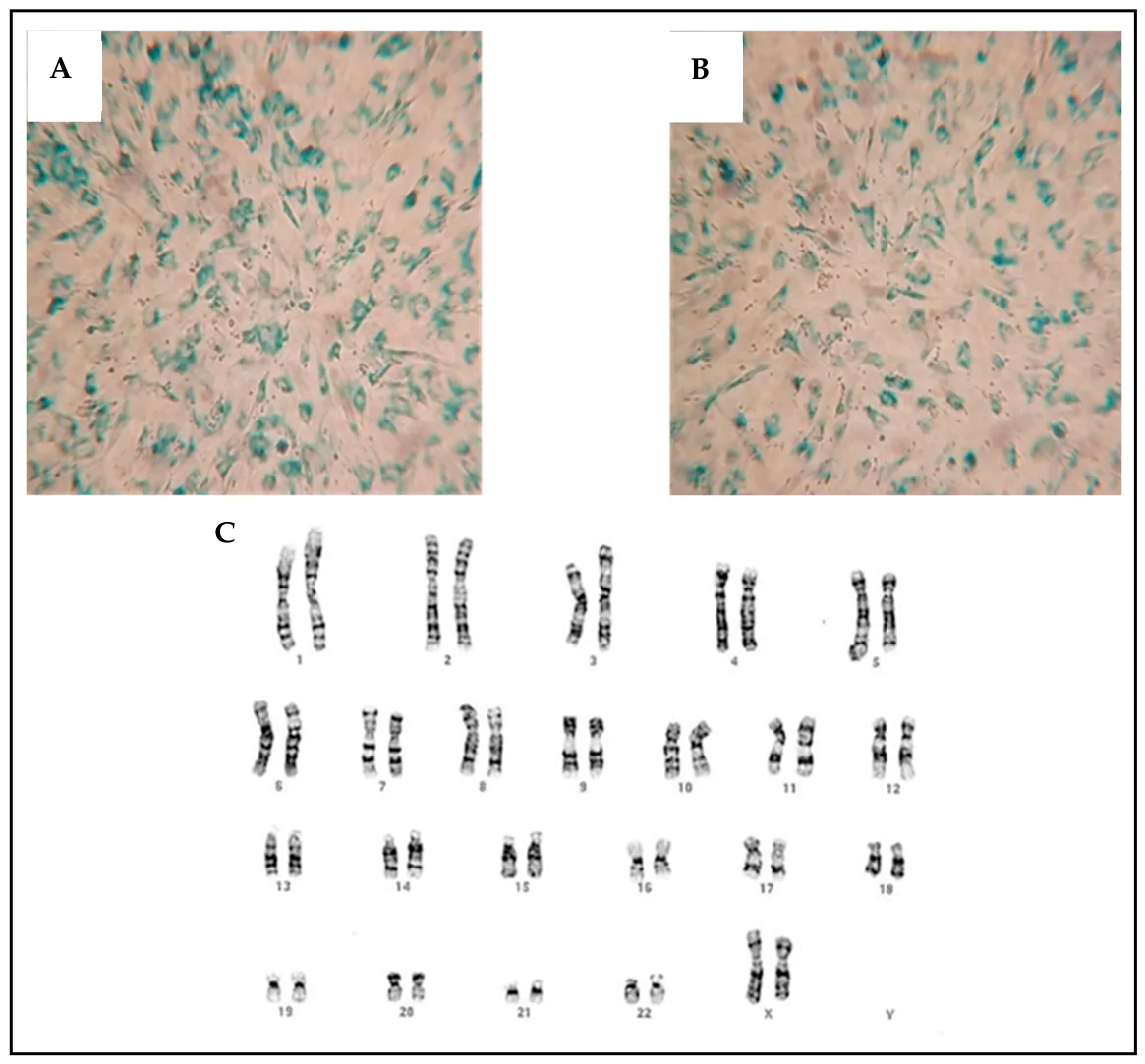
Senescence and conventional karyotype of a representative MSC line. MSCs exposed (**A**) and non-exposed (**B**) to TMR^®^ entered senescence at P9. (**C**) Karyotyping in a TMR-exposed culture. No chromosomal aberrations are present.

TMR did not influence MSCs in reaching senescence passages; exposed and non-exposed cell lines entered senescence at passage 9 (P9), showing that they did not acquire tumorigenic transformation. This observation is also confirmed by cytogenetic analysis, which did not show any chromosomal aberration by conventional karyotyping in TMR-exposed cultures (Figure 1).

### 3.2. Proliferation Assay

**Figure 2:**
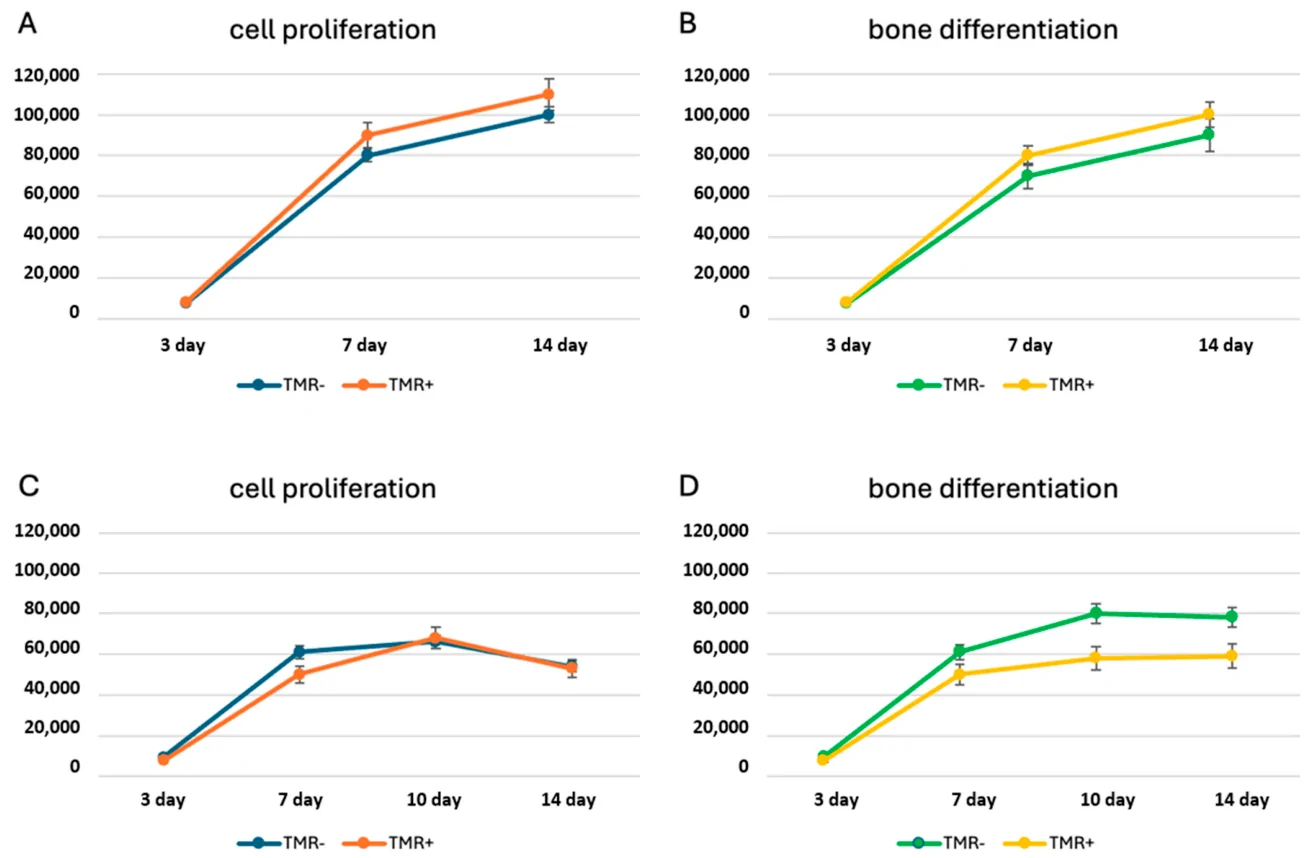
BM-MSC (**A**) and AD-MSC (**C**) growth curves at 3, 7, and 14 days in complete medium exposed to TMR (orange line) and non-exposed (blue line). BM-MSCs (**B**) and AD-MSCs (**D**) differentiation curves at 3, 7, and 14 days with differentiation medium exposed to TMR (yellow line) and non-exposed (green line).

We considered four BM-MSCs and two AD-MSCs lines, performing four technical replications for each line. MSC growth evaluated by the Alamar Blue assay showed similar behavior between exposed and non-exposed cultures. Moreover, the growth evaluation after *in vitro* osteogenic induction showed a reduction compared to non-induced MSCs, showing that the synergy between biological and physical factors contributes positively to the progression of bone differentiation (Figure 2).

### 3.3. Bone Differentiation Evaluation

qRT-PCR analysis was conducted on TMR-exposed and non-exposed MSCs.

The specificity of our PCR reaction was ensured by the presence of a negative control (working mix without cDNA) for each primer and for each run. Moreover, the melt curve plot showed only one peak corresponding to a single amplicon, which was not present in the negative control.

The genes analyzed were expressed as fold change versus the gene expression of MSC cultured in the absence of osteogenic medium (control).

In exposed cultures, Runx2 expression was increased at 3 and 7 days for BM-MSCs and decreased at 14 days compared to unexposed cells. In AD-MSCs, the expression trend was comparable but shifted in time, and for this reason, AD-MSCs were not analyzed for this gene expression at 3 days. The expression was slower during the period of culture for both cell lines.

ALP gene expression was increased at 3 and 7 days in exposed BM-MSCs, with a decrease during the culture. While an opposite trend was observed in AD-MSCs.

**Figure 3:**
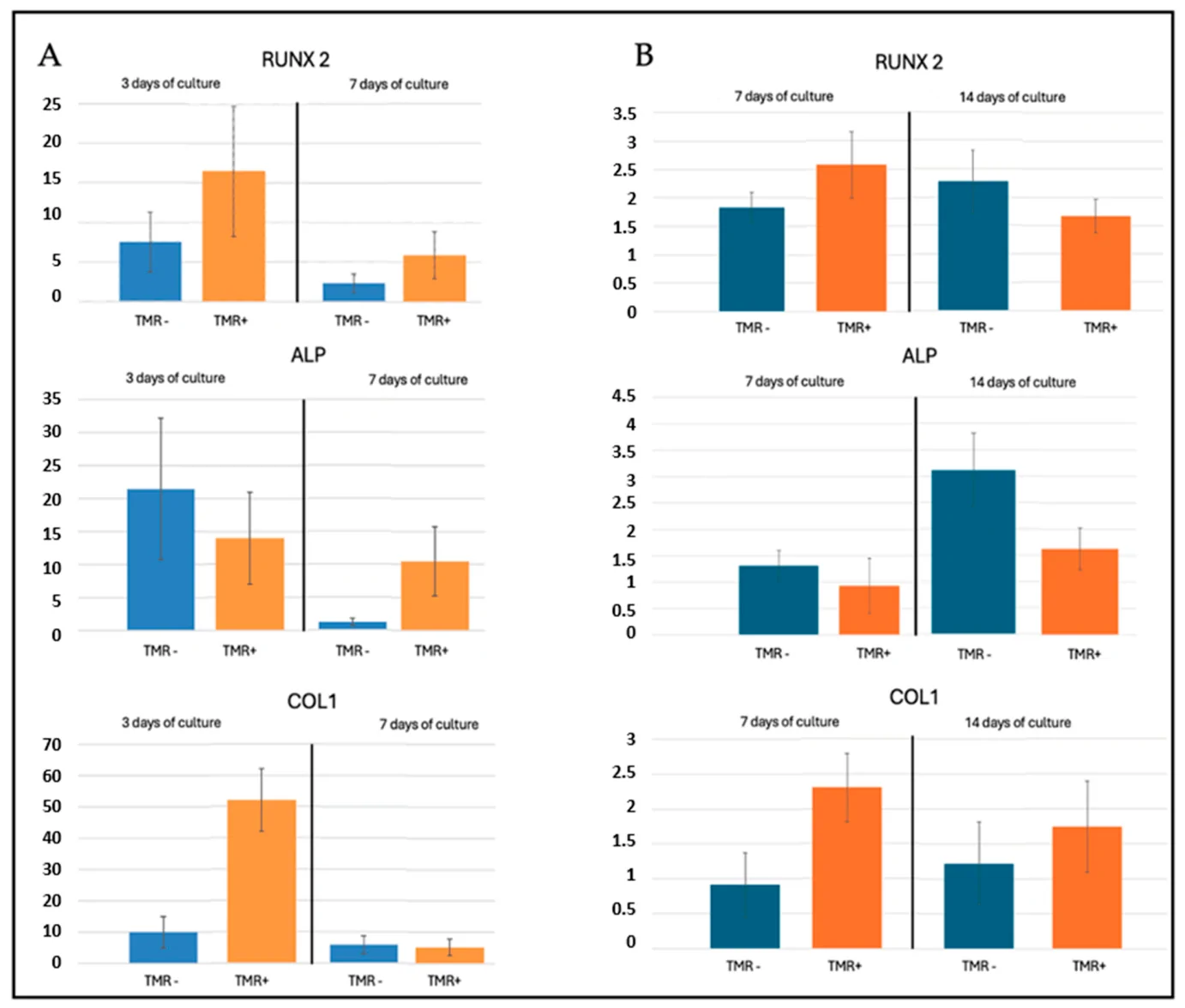
(**A**) Gene expression of BM-MSCs cultured in osteogenic medium exposed to TMR (orange box) and non-exposed (blue box) for 3 and 7 days. (**B**) Gene expression of AD-MSCs cultured in osteogenic medium exposed to TMR (orange box) and non-exposed (blue box) for 7 and 14 days.

Exposed BM-MSCs exhibited an increase in COL gene expression at 3 and 7 days, with a decrease at 14 days. For AD-MSCs, we observed an increase in gene expression at 7 and 14 days compared to unexposed ones (Figure 3).

**Figure 4:**
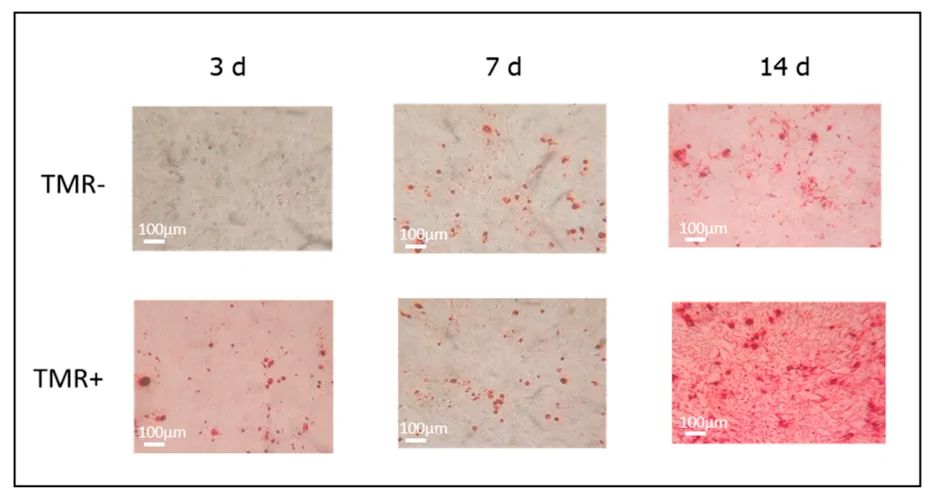
Alizarin Red S staining on MSCs exposed or not to TMR for 3, 7 and 14 days.

**Figure 5:**
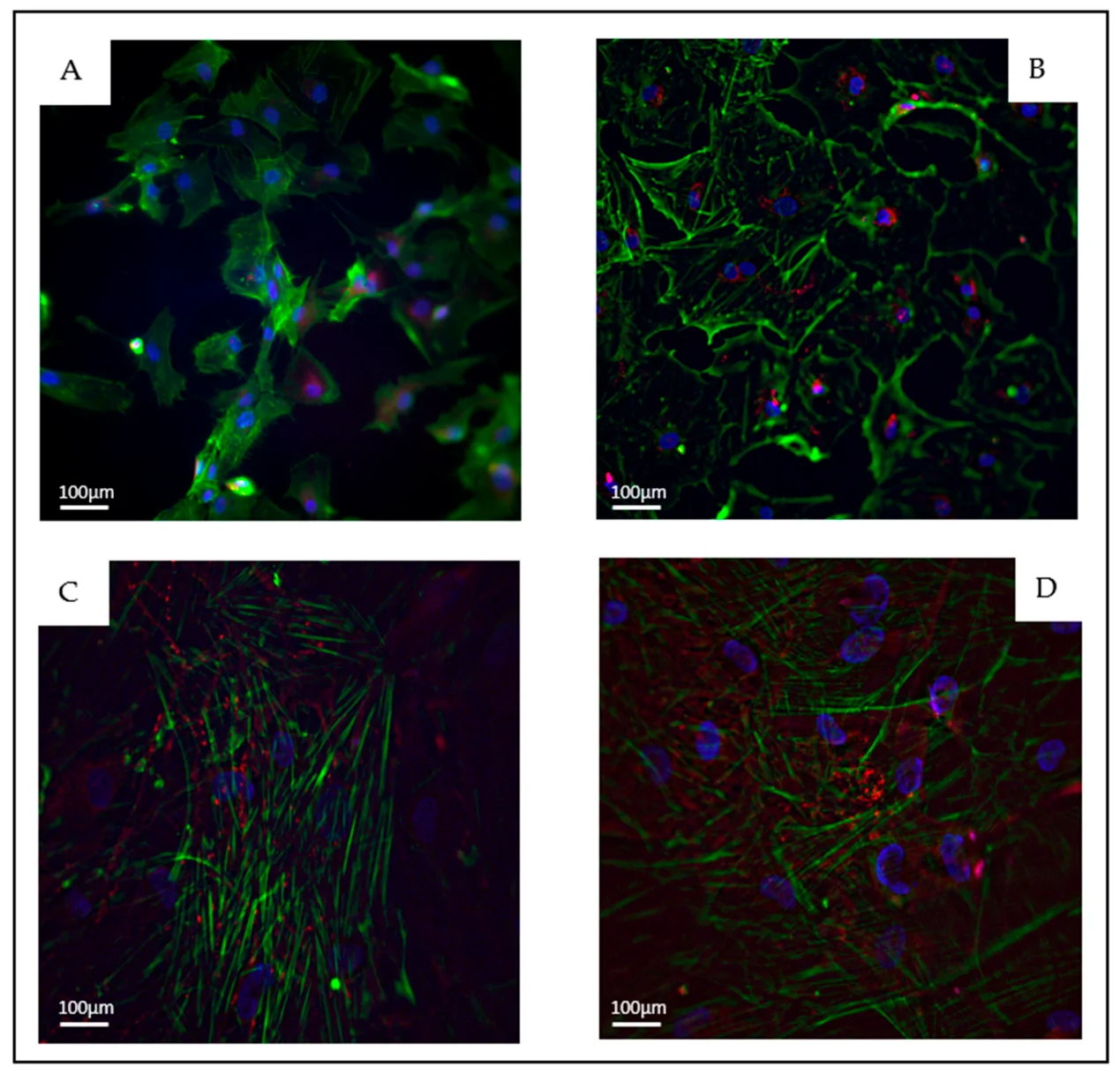
Immunofluorescence assay of AD-MSCs after 21 days of differentiation. (**A**) AD-MSCs cultured in growth medium non-exposed to TMR (**A**), exposed to TMR (**B**), (**C**) AD-MSCs cultured in osteogenic medium non-exposed to TMR (**C**), and exposed to TMR (**D**). The cell nuclei (DAPI) are marked in blue, the cytoskeleton (phalloidin) is marked in green, while the collagen (COL1A1) is marked in red.

Even if the reported data did not result in statistically different results, they were confirmed for BM-MSCs by histological staining of calcium deposition (Figure 4) and for AD-MSCs by immunofluorescence assay (Figure 5).

The presence of collagen deposition was evaluated in AD-MSCs before and after osteogenic induction, in exposed and non-exposed cultures.

We observed a greater deposition of collagen in MSCs exposed than in non-exposed ones, both in the presence and absence of osteogenic medium.

### 3.4. In Vitro Immunomodulatory Effect

We tested the influence of TMR on the MSC *in vitro* immunomodulatory capacities on PHA-activated PBMC proliferation. MSC inhibition did not show changes between exposed or non-exposed co-cultures even at different ratios PBMC: MSCs.

**Figure 6:**
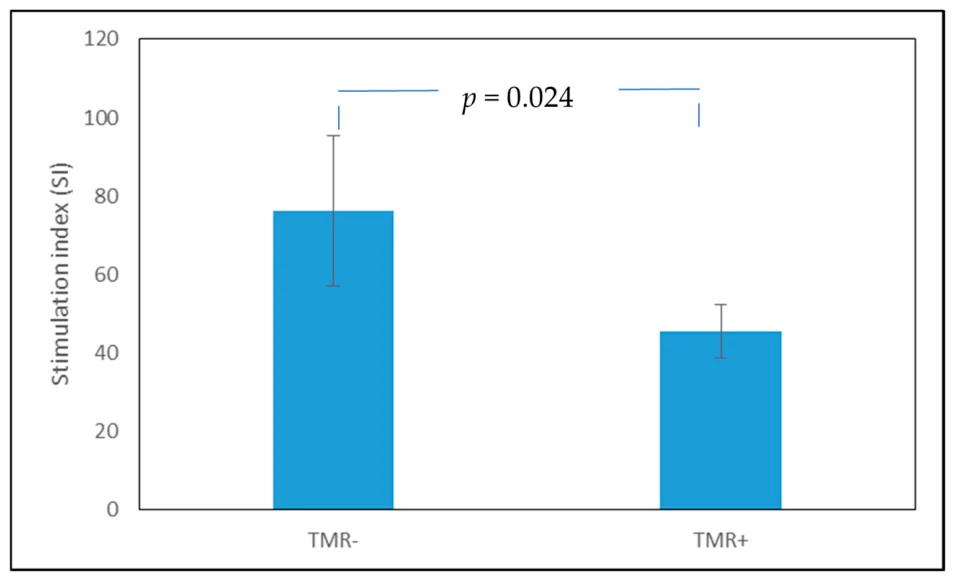
In vitro TMR immunomodulatory effect on PBMCs activated with PHA. Results are expressed as a stimulation index (SI = cpm stimulated/cpm unstimulated).

Of interest, we observed that PBMC proliferation was significantly lower (*p* = 0.024) in TMR-treated (45.6 ± 6.8) than in non-exposed PBMCs (76.3 ± 19.1) (Figure 6).

**Figure 7:**
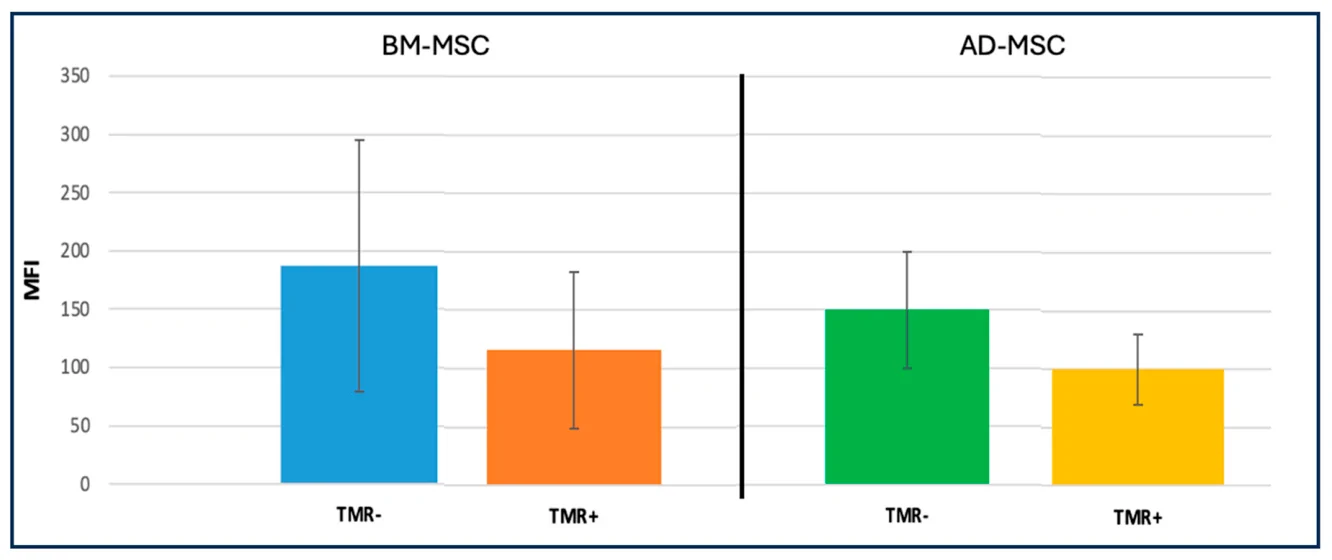
ROS evaluation in exposed and non-exposed MSCs (results are reported as MFI). Blue and orange: BM-MSCs; green and yellow: AD-MSCs.

We tested the influence of TMR on reactive oxygen species (ROS) production, and we observed a reduction in ROS in MSCs after TMR^®^ exposure without significant differences (Figure 7).

## 4. Discussion

In recent years, several studies have shown that pulsed electromagnetic fields (PEMFs) represent a well-established, non-invasive therapeutic support [23,24,25]. By influencing proliferation, differentiation, and the secretion of trophic factors, PEMFs interact with the cellular processes involved in bone and cartilage regeneration. The application of PEMFs can reduce joint pain, both chronic and postoperative, and play a significant role in repairing bone, cartilage, and connective tissue and in reducing cellular inflammation and swelling with an improved functional recovery [26,27]. Recently, the new technology (TMR^®^), characterized by the use of a range of biophysical frequencies validated to act directly on the bioelectric component of the cell, has been proposed for clinical application.

In this *in vitro* study, we evaluated the effect of TMR^®^ on stromal cells derived from bone marrow and adipose tissue (BM-MSCs and AD-MSCs).

First of all, our results showed that TMR^®^ did not induce tumorigenic transformation in exposed MSCs. This is demonstrated by conventional karyotyping, maintenance of the capacity to enter senescence and normal proliferation, together with preservation of the specific antigen surface expression.

It is reported that the PEMFs influence several molecular pathways (including TGF-β/BMP, PI3K/Akt/mTOR, Notch, and MAPK/ERK), stimulating the expression of osteogenic markers (RUNX-2, ALP) and promoting the formation of mineralized matrix [28,29].

Of interest, we observed that TMR^®^ exposed MSCs exhibit an earlier osteogenic differentiation ability compared to non-exposed counterparts, with higher expression of osteogenic genes such as *runx-2*, *alp*, and *Coll1A1*.

Runx2 is an early marker of bone differentiation, being the first transcription factor activated during differentiation in the osteoblast lineage, decreasing when osteoblasts become mature [30]. Alp plays a role in the first phase of differentiation and in bone mineralization, representing a marker of early and late differentiation [31], while Col1a1 is associated with osteogenic differentiation and mineralization [27].

In the present study, we observed that exposed BM-MSCs showed *Col1a1* gene expression very early during culture. While exposed AD-MSCs exhibited collagen deposition, demonstrated by immunofluorescence, even without osteogenic medium addition, suggesting that TMR^®^ may induce, at least in AD-MSCs, slight differentiation without adding osteogenic stimuli. This is of great interest with a view to clinical application in bone repair since complete fracture healing occurs during the bone remodeling phase, in which mature osteoblasts deposit collagen and calcium at the fracture site, allowing the formation of the bone callus.

Recent data demonstrate that electromagnetic fields reduce the inflammatory response by activating adenosine receptors, resulting in inhibition of the NF-κB pathway [26,32]. We also observed that TMR^®^ treatment induced the inhibition of *in vitro* lymphocyte activation, suggesting an anti-inflammatory effect of this technology as well as a reduction of reactive oxygen species (ROS), whose production may lead to a delay and impairment of the healing process [33]. Our observations of MSCs after TMR^®^ exposure may indicate this technology as a possible device for clinical applications and for *in vitro* modulation of MSC properties.

In agreement with previously reported data on PEMFs, and on the basis of our *in vitro* observations, we hypothesize that *in vivo* TMR^®^ may act during the different phases of fracture healing involving MSC recruitment and differentiation.

However, further studies are needed to confirm our *in vitro* results, and clinical trials should be designed to translate TMR^®^ technology to different clinical applications beyond the orthopedic field. We are aware that our results are exploratory, but we believe that they could provide some more data to better understand the cellular response induced by TMR.

## 5. Conclusions

Our results suggest that TMR^®^ may contribute to the bone healing process by stimulating stem cell activity, by enhancing the expression of bone-related genes, and by promoting tissue formation. It appears safe since it does not induce tumorigenic changes in MSCs, preserving their properties. In conclusion, TMR^®^ could be a good non-invasive therapeutic treatment in clinical applications for orthopedic recovery.

## Figures and Tables

**Table 1:** Primers used in real-time PCR experiments.

Gene	Target Transcript	Quantitect Primer Assay (Qiagen)	Amplicon Length
*ALP*	NM_000478	QT00012957	110 bp
*COL1A1*	NM_000088	QT00037793	127 bp
*RUNX-2*	NM_001015051	QT00020517	102 pb
*β2M*	NM_004048	QT00088935	98 pb

**Table 2:** Cell surface markers of BM- and AD-MSCs. Results are expressed as percentage of gated cells. TMR− = non-exposed; TMR+ = exposed.

	BM-MSCs (% of Expression)		AD-MSCs (% of Expression)	
Cell Surface Markers (CD)	TMR−	TMR+	TMR−	TMR+
CD73	100	100	98	98
CD34	0	0	0	0
CD90	100	100	99	99
CD14	0	0	0	0
CD45	0	0	0	0
CD105	100	100	99	99
HLA-DR	0	0	0	0

## Data Availability

The original contributions presented in this study are included in the article. Further inquiries can be directed to the corresponding author.

## References

[1] Fukada E., Yasuda I. (1957). On the Piezoelectric Effect of Bone. J. Phys. Soc. Jpn..

[2] Douglas W.W. (1963). A Possible Mechanism of Neurosecretion: Release of Vasopressin by Depolarization and Its Dependence on Calcium. Nature.

[3] Bassett C.A.L. (1982). Pulsing Electromagnetic Fields: A New Method to Modify Cell Behavior in Calcified and Noncalcified Tissues. Calcif. Tissue Int..

[4] Caliogna L., Bina V., Brancato A.M., Gastaldi G., Annunziata S., Mosconi M., Grassi F.A., Benazzo F., Pasta G. (2022). The Role of PEMFs on Bone Healing: An In Vitro Study. Int. J. Mol. Sci..

[5] Li S., Yu B., Zhou D., He C., Zhuo Q., Hulme J.M. (2013). Electromagnetic Fields for Treating Osteoarthritis. Cochrane Database Syst. Rev..

[6] Hannemann P.F.W., Mommers E.H.H., Schots J.P.M., Brink P.R.G., Poeze M. (2014). The Effects of Low-Intensity Pulsed Ultrasound and Pulsed Electromagnetic Fields Bone Growth Stimulation in Acute Fractures: A Systematic Review and Meta-Analysis of Randomized Controlled Trials. Arch. Orthop. Trauma Surg..

[7] Piotrzkowska D., Siwak M., Adamkiewicz J., Dziki L., Majsterek I. (2025). The Therapeutic Potential of Pulsed Electromagnetic Fields (PEMF) and Low-Intensity Pulsed Ultrasound (LIPUS) in Peripheral Nerve Regeneration: A Comprehensive Review. Int. J. Mol. Sci..

[8] Wang H., Zou W., Cao Y. (2024). Bioelectromagnetic Fields as Signaling Currents of Life. Radiat. Med. Prot..

[9] Zhao M. (2009). Electrical Fields in Wound Healing—An Overriding Signal That Directs Cell Migration. Semin. Cell Dev. Biol..

[10] Aziz Z., Flemming K., The Cochrane Collaboration (2012). Electromagnetic Therapy for Treating Pressure Ulcers. Cochrane Database of Systematic Reviews.

[11] Novickij V., Grainys A., Lastauskienė E., Kananavičiūtė R., Pamedytytė D., Kalėdienė L., Novickij J., Miklavčič D. (2016). Pulsed Electromagnetic Field Assisted in Vitro Electroporation: A Pilot Study. Sci. Rep..

[12] Perrier D.L., Rems L., Boukany P.E. (2017). Lipid Vesicles in Pulsed Electric Fields: Fundamental Principles of the Membrane Response and Its Biomedical Applications. Adv. Colloid Interface Sci..

[13] Daish C., Blanchard R., Fox K., Pivonka P., Pirogova E. (2018). The Application of Pulsed Electromagnetic Fields (PEMFs) for Bone Fracture Repair: Past and Perspective Findings. Ann. Biomed. Eng..

[14] Siwak M., Piotrzkowska D., Skrzypek M., Majsterek I. (2024). Effects of PEMF and LIPUS Therapy on the Expression of Genes Related to Peripheral Nerve Regeneration in Schwann Cells. Int. J. Mol. Sci..

[15] Caliogna L., Medetti M., Bina V., Brancato A.M., Castelli A., Jannelli E., Ivone A., Gastaldi G., Annunziata S., Mosconi M. (2021). Pulsed Electromagnetic Fields in Bone Healing: Molecular Pathways and Clinical Applications. Int. J. Mol. Sci..

[16] Friedenstein A.J., Chailakhjan R.K., Lalykina K.S. (1970). The Development of Fibroblast Colonies in Monolayer Cultures of Guinea-Pig Bone Marrow and Spleen Cells. Cell Prolif..

[17] Dominici M., Le Blanc K., Mueller I., Slaper-Cortenbach I., Marini F.C., Krause D.S., Deans R.J., Keating A., Prockop D.J., Horwitz E.M. (2006). Minimal Criteria for Defining Multipotent Mesenchymal Stromal Cells. The International Society for Cellular Therapy Position Statement. Cytotherapy.

[18] Valsecchi C., Croce S., Lenta E., Acquafredda G., Comoli P., Avanzini M.A. (2023). New Therapeutic Approaches in Pediatric Diseases: Mesenchymal Stromal Cell and Mesenchymal Stromal Cell-Derived Extracellular Vesicles as New Drugs. Pharmacol. Res..

[19] Ferroni L., Bellin G., Emer V., Rizzuto R., Isola M., Gardin C., Zavan B. (2015). Treatment by Therapeutic Magnetic Resonance (TMR^TM^) Increases Fibroblastic Activity and Keratinocyte Differentiation in an in Vitro Model of 3D Artificial Skin: Magnetic Resonance Increases Keratinocyte Organization. J. Tissue Eng. Regen. Med..

[20] Shaffer L.G., A Rosenfeld J. (2013). Microarray-based prenatal diagnosis for the identification of fetal chromosome abnormalities. Expert Rev. Mol. Diagn..

[21] Conforti A., Scarsella M., Starc N., Giorda E., Biagini S., Proia A., Carsetti R., Locatelli F., Bernardo M.E. (2014). Microvescicles Derived from Mesenchymal Stromal Cells Are Not as Effective as Their Cellular Counterpart in the Ability to Modulate Immune Responses In Vitro. Stem Cells Dev..

[22] Jing F., Wang Q., Xu Y., Dong J., Huang H., Li Y. (2024). MAZ Regulates Ferroptosis, Apoptosis and Differentiation of Oligodendrocyte Precursor Cells. Brain Res..

[23] Carbone I.F., Gigli F.M.P., Rossi G., Romagnoli V., Gallicola B., Sandi F., Esposito G., Ferrazzi E.M. (2024). Pulsating Electromagnetic Fields for Perineal Lacerations and Surgical Wounds Healing in the Postpartum: A Pilot Study. Arch. Gynecol. Obstet..

[24] Ferroni L., Gardin C., De Pieri A., Sambataro M., Seganfreddo E., Goretti C., Iacopi E., Zavan B., Piaggesi A. (2017). Treatment of Diabetic Foot Ulcers with Therapeutic Magnetic Resonance (TMR^®^) Improves the Quality of Granulation Tissue. Eur. J. Histochem..

[25] Abbruzzese L., Iacopi E., Coppelli A., Bonino G., Goretti C., Piaggesi A. (2015). Safety and Effectiveness of Therapeutic Magnetic Resonance in the Management of Postsurgical Lesion of the Diabetic Foot. Int. J. Low. Extrem. Wounds.

[26] Massari L., Benazzo F., Falez F., Perugia D., Pietrogrande L., Setti S., Osti R., Vaienti E., Ruosi C., Cadossi R. (2019). Biophysical Stimulation of Bone and Cartilage: State of the Art and Future Perspectives. Int. Orthop..

[27] Huang W., Yang S., Shao J., Li Y.P. (2007). Signaling and transcriptional regulation in osteoblast commitment and differentiation. Front. Biosci..

[28] Yuan J., Xin F., Jiang W. (2018). Underlying Signaling Pathways and Therapeutic Applications of Pulsed Electromagnetic Fields in Bone Repair. Cell. Physiol. Biochem..

[29] Zhang B., Xie Y., Ni Z., Chen L. (2020). Effects and Mechanisms of Exogenous Electromagnetic Field on Bone Cells: A Review. Bioelectromagnetics.

[30] Liu J.C., Lengner C.J., Gaur T., Lou Y., Hussain S., Jones M.D., Borodic B., Colby J.L., Steinman H.A., Van Wijnen A.J. (2011). Runx2 Protein Expression Utilizes the Runx2 P1 Promoter to Establish Osteoprogenitor Cell Number for Normal Bone Formation. J. Biol. Chem..

[31] Pham Q.P., Kasper F.K., Baggett L.S., Raphael R.M., Jansen J.A., Mikos A.G. (2008). The influence of an in vitro generated bone-like extracellular matrix on osteoblastic gene expression of marrow stromal cells. Biomaterials.

[32] Ferroni L., Gardin C., Dolkart O., Salai M., Barak S., Piattelli A., Amir-Barak H., Zavan B. (2018). Pulsed Electromagnetic Fields Increase Osteogenetic Commitment of MSCs via the mTOR Pathway in TNF-α Mediated Inflammatory Conditions: An in-Vitro Study. Sci. Rep..

[33] Ehnert S., Fentz A.-K., Schreiner A., Birk J., Wilbrand B., Ziegler P., Reumann M.K., Wang H., Falldorf K., Nussler A.K. (2017). Extremely Low Frequency Pulsed Electromagnetic Fields Cause Antioxidative Defense Mechanisms in Human Osteoblasts via Induction of •O_2_^−^ and H_2_O_2_. Sci. Rep..

